# Hunting the Extinct Steppe Bison (*Bison priscus*) Mitochondrial Genome in the Trois-Frères Paleolithic Painted Cave

**DOI:** 10.1371/journal.pone.0128267

**Published:** 2015-06-17

**Authors:** Marie-Claude Marsolier-Kergoat, Pauline Palacio, Véronique Berthonaud, Frédéric Maksud, Thomas Stafford, Robert Bégouën, Jean-Marc Elalouf

**Affiliations:** 1 iBiTec-S/SBiGeM, CEA/Saclay 91191 Gif-sur-Yvette cedex, France; 2 CNRS-UMR7206, Eco-anthropologie et Ethnobiologie, Département Hommes, Natures et Sociétés, Musée de l’Homme, 17 place du Trocadéro, 75016 Paris, France; 3 Service Régional de l’Archéologie, 32 rue de la Dalbade, BP811 31080 Toulouse cedex 6, France; 4 AMS 14C Dating Centre, Department of Physics and Astronomy, University of Aarhus, Ny Munkegade 120, Aarhus, Denmark; 5 Association Louis Bégouën, Laboratoire de Préhistoire de Pujol, 09200 Montesquieu-Avantès, France; Natural History Museum of Denmark, University of Copenhagen, DENMARK

## Abstract

Despite the abundance of fossil remains for the extinct steppe bison (*Bison priscus*), an animal that was painted and engraved in numerous European Paleolithic caves, a complete mitochondrial genome sequence has never been obtained for this species. In the present study we collected bone samples from a sector of the Trois-Frères Paleolithic cave (Ariège, France) that formerly functioned as a pitfall and was sealed before the end of the Pleistocene. Screening the DNA content of the samples collected from the ground surface revealed their contamination by *Bos* DNA. However, a 19,000-year-old rib collected on a rock apart the pathway delineated for modern visitors was devoid of such contaminants and reproducibly yielded *Bison priscus* DNA. High-throughput shotgun sequencing combined with conventional PCR analysis of the rib DNA extract enabled to reconstruct a complete mitochondrial genome sequence of 16,318 bp for the extinct steppe bison with a 10.4-fold coverage. Phylogenetic analyses robustly established the position of the *Bison priscus* mitochondrial genome as basal to the clade delineated by the genomes of the modern American *Bison bison*. The extinct steppe bison sequence, which exhibits 93 specific polymorphisms as compared to the published *Bison bison* mitochondrial genomes, provides an additional resource for the study of *Bovinae* specimens. Moreover this study of ancient DNA delineates a new research pathway for the analysis of the Magdalenian Trois-Frères cave.

## Introduction

The earliest members of the genus *Bison* appeared at the beginning of the Pleistocene in India and China. Bison then spread from Asia to Europe and America. Archaeological evidence shows that two bison species existed in Europe from the Middle Pleistocene on, the steppe bison and the woodland bison. The steppe bison, *Bison priscus* (Bojanus, 1827), is very common in Pleistocene deposits but became extinct at the end of the last Ice Age, about 10,000 years ago. It was a formidable animal with long horns and robust legs that may have stood more than 2 meters at the withers and reached a total length of more than 2.7 meters. It occupied cool, steppe-like grasslands. *Bison priscus* had a wide geographic distribution, called the great Pleistocene bison belt, which extended from England to America and from the archipelago of Nova Zembla in the Arctic Ocean in the north of Russia down to Spain, Caucasia and Mexico. It is generally reckoned that it is the steppe bison that was portrayed by paleolithic artists in painted caves like the Altamira cave in northern Spain, the Chauvet, Lascaux and Trois-Frères caves in France. The woodland bison, *Bison schoetensacki* (Freudenberg, 1910), which has left more scarce fossil remains than the steppe bison, appeared in the early Middle Pleistocene. Its body size and horns were smaller than those of the steppe bison and it was probably a forest-dweller. It became extinct at the end of the Pleistocene at the latest [1].

Only two bison species survive today: the American bison and the European bison. The American bison, in North America, includes two subspecies, the plains bison, *Bison bison bison*, and the wood bison, *Bison bison athabascae*. The European bison, *Bison bonasus*, is found in Europe and the Caucasus, where it has been reintroduced after its extinction as a wild species. The phylogenetic relationships between the extant and the extinct species are a matter of debate. A population of *Bison priscus* is believed to have first entered eastern Beringia from Asia during the middle Pleistocene, around 300 to 130 ky ago, and then moved southward into central North America approximately 130 to 75 ky ago [2]. The analysis of mitochondrial sequences of bison specimens from Siberia, China and North America (dated between present time and about 70 ky B.P. [before present]) has suggested that late Pleistocene bison from the Ural mountains to northern China are descendants of one or more dispersals from North America [2]. This study has also indicated gene flow between bison populations in Beringia and central North America between 60 and 25 ky B.P., before ice sheet formation during the Last Glacial Maximum (22 to 18 ky B.P.) hampered north-south faunal exchange [2]. The retreat of ice sheets around 14 ky B.P. created an ice-free corridor through which dispersal between Beringia and North America could occur. However, genetic exchanges between bison in Beringia and in central North America were soon after limited due to the establishment of spruce forest in Alberta and of peatland across western and northwestern Canada. As a result, all modern *Bison bison* belong to a clade distinct from Beringian bison [2]. The European bison *Bison bonasus* has a complex descent: its nuclear genome is closely related to that of *Bison bison*, with which it can produce completely fertile hybrid offspring, but its mitochondrial genome is more similar to the genomes of species belonging to the *Bos* genus than to the genome of *Bison bison* [3]. These observations could be explained by lineage sorting implying an early divergence of the American and European bison mitochondrial sequences, or by a scenario according to which the nuclear DNA of an ancient Eurasian *Bos* population would have been changed by the systematic introgression of *Bison* bulls (either *Bison priscus*, *Bison bison* migrating back to Europe, or *Bison schoetensacki*). This ancient hybridization would have been facilitated by the social structure of herd species, in which most males are excluded from reproduction by the dominating bulls, and would have created a new species with a nuclear genome of bison type, but with a *Bos*-like mitochondrial DNA from the maternal ancestor. Interestingly, this scenario would explain the sudden paleontological appearance of *Bison bonasus* at the Preboreal stage (10.3 to 9 ky B.P.) [3].

In an effort to investigate the evolution and demographic history of Pleistocene bison, partial sequences of *Bison priscus* mitochondrial genome have already been obtained [2,4]. The mitochondrial control region, also called the D-loop region, has been sequenced in ~ 300 *Bison priscus* fossils collected in Siberia, China and North America [2,4]. However, these partial sequences are a few hundred bp long, representing less than 5% of the expected 17-kb mitochondrial genome. The purpose of the present study was to fill the gap by providing a complete mitochondrial genome sequence for the extinct steppe bison.

This work presents the analysis of several bone samples that were collected from the Trois-Frères cave (Ariège, France). This cave (43° 1′ N, 1° 12′ E) was named after the three sons of Comte Henri Bégouën who discovered its entrance in July 1914. It contains numerous animal representations made famous by the publication of the Abbé Henri Breuil [5], including the drawing and engraving of at least 170 bison. The importance of the bison for the Magdalenian artists who decorated the cave is obvious not only from the fact that its representations outnumber those of any other species, but also from the drawing of a therianthropic figure with a bison upper part. Besides, the cave of the Trois-Frères is part of a single cave-complex with the Tuc d'Audoubert, which contains two modeled clay bison, a unique masterpiece of the Paleolithic period [6]. DNA extracted from one rib bone sample was sequenced using Illumina technology which, in combination with conventional PCR and Sanger sequencing, allowed the assembly of a complete sequence of *Bison priscus* mitochondrial genome.

## Material and Methods

### Collection of archeological samples

All necessary permits were obtained for the described study, which complied with all relevant regulations. The four samples analyzed in this study correspond to specimens SGE1, SGE2, SGE3 and SGE5 of the Trois-Frères cave (Montesquieu-Avantès, 09200, France). They were collected under the supervision of two of us (RB, FM). The samples are stored in the Museum of the Association Louis Bégouën in Montesquieu-Avantès. The permit to collect the samples and perform their analysis was provided by the Direction Régionale des Affaires Culturelles Midi-Pyrénées, Service Régional de l’Archéologie (32, rue de la Dalbade, BP811, 31080 Toulouse Cedex 6, France) where it is registered under the reference FM/MB/2013/13481.

### Contamination issues

To avoid contamination derived from previous and current analyses, initial steps (*i*.*e*. ancient DNA extraction, generation of the DNA library, and set-up of PCR reactions) were carried out in a dedicated laboratory located in a building where no molecular work has ever been performed on extant DNA. Access to this building was not allowed to persons who had worked on the same day in the modern DNA laboratory and rested on wearing dedicated shoes and laboratory coats. These were changed for a second pair of shoes and a single-use laboratory coat for working in the ancient DNA laboratory. Gloves were changed frequently. Negative controls included mock extracts and PCR blanks (where water was added instead of DNA extracts), which always failed to yield any amplification product. To screen further for the possibility of contaminants, we aligned the Illumina reads of the DNA library to the reference mitochondrial genome sequence of *Homo sapiens* (GenBank accession number NC_012920.1 [7]) using BWA version 0.5.7 [8]. Out of 1 billion DNA reads, we found 11 reads with a minimal length of 30 bp that perfectly matched the mitochondrial genome sequence of *Homo sapiens*, five of which also perfectly matched the reference mitochondrial genome of *Bison bison* (GenBank accession number NC_012346 [9]). By contrast, the same alignment parameters yielded 1,825 reads that perfectly matched the *Bison bison* reference mitochondrial genome sequence.

### DNA extraction

The bone surface was scraped to remove deposits, and 0.5 g of cortical bone powder was produced using a rotary drill. Bone powder was incubated 40 h at 42°C under constant agitation in 5 ml of extraction buffer consisting of 0.45 M EDTA, 10 mM Tris-HCl (pH 8.0), 0.1% SDS, 65 mM DTT, and 0.5 mg/ml proteinase K. After centrifugation, the supernatant was recovered, extracted once with one volume of phenol, once with a phenol-chloroform (50:50) mixture, and once with chloroform. The aqueous phase was then dialyzed and concentrated using Amicon ultra 2 ml centrifugal filter device with a cutoff of 30 kD (Millipore, Billerica, MA), the column was washed 4-times with distilled water, and the DNA extract was recovered in a volume of 100 to 120 μl. It was purified further using a silica-membrane-based method designed for small DNA fragments (Qiagen purification kit #28004; Venlo, Netherlands). The final extract consisted of a sample of 100 μl.

### PCR screening of the samples for bison DNA content

Screening of the bone samples was performed by PCR. All four samples were analyzed using primer pair 1 (S1 Table). Then, the most promising sample (SGE2) was further studied using primer pairs 2 to 10. PCR was performed in a 50-μl reaction volume containing 1 μl of mock or ancient DNA extracts, 300 pM of sense and antisense primers, 200 μM dNTP, 2.5 mM MgCl_2_, 5 μl of GeneAmp 10X PCR buffer II, and 2.5 U of AmpliTaq Gold DNA polymerase (Thermo Fisher Scientific, Waltham, MA, USA). Blank samples (containing water instead of bone or mock extracts) were also included in each PCR experiment. Only bone extracts yielded amplification products. After an activation step (95°C, 8.5 min), a single round of 45 PCR cycles (95°C, 15 sec; 50–60°C (according to primers *Tm*), 20 sec; 70°C, 1 min) was performed in a Veriti thermal cycler (Thermo Fisher Scientific). The full reaction volume was loaded onto an 8% polyacrylamide gel stained with Sybr Green I (Thermo Fisher Scientific). PCR amplicons were eluted from the gel and inserted into pCR4-TOPO (Thermo Fisher Scientific). Each PCR amplicon was characterized by sequencing 3 to 33 cloned DNA fragments using M13 forward primer.

### Generation and sequencing of a library of DNA fragments

A library of DNA fragments suitable for high-throughput sequencing with the Illumina procedure [10] was generated from 5 μl of the SGE2 DNA extract 2 using the Illumina DNA sample prep kit (PE-102-1001). We followed the manufacturer’s recommendations (San Diego, CA, USA), except for modifications described elsewhere [11] that were introduced for the purpose of analyzing ancient DNA. Briefly, blunt-end DNA fragments were produced using T4 and Klenow DNA polymerases, and 5’ ends were phosphorylated using T4 polynucleotide kinase. A deoxyadenosine was added to the 3’ ends of the fragments using Klenow exo^-^ DNA polymerase and dATP to prevent the DNA fragments from ligating to one another during the adapter ligation reaction. Ligation to Illumina adapters was then performed using DNA ligase and one tenth of the amount of adapters recommended by the manufacturer.

Amplification of the library was performed using Phusion DNA polymerase. Twelve PCR cycles were enough to produce sufficient amounts of DNA for sequencing.

DNA sequencing was performed at Genoscope (Evry, France) on Illumina HiSeq 2000 (3 sequencing lanes) and HiSeq 2500 platforms (4 sequencing lanes) using HiSeq v3 chemistry with a read length set to 101 nucleotides and analysis on the single read mode.

### Illumina sequencing data analysis

Reads were trimmed for adapter sequences, N’s and low quality stretches on the 3’ end, using a software based on the FASTX-Toolkit package (http://hannonlab.cshl.edu/fastx_toolkit) and designed by Genoscope. After this step, sequences shorter than 20 nucleotides were discarded using an in-house Python script, yielding a dataset of 1,033,460,536 DNA reads (543,346,926 reads from the HiSeq 2000 sequencing lanes, and 490,113,610 reads from the HiSeq 2500 sequencing lanes). The DNA reads of this study have been deposited at EBI under accession number ERP006376.

### Assembling the *Bison priscus* mitochondrial genome sequence

The sequence assembly was performed in three steps. First the Illumina reads ranging in size between 20 and 101 nucleotides were aligned to the mitochondrial genome of *Bison bison* and to the mitochondrial control region of *Bison priscus* isolate BS212 (GenBank accession number AY748539.1, [2]) using BWA with default options except for-*o* (gap opening) and-*n* (maximum edit distance), which were set to 0 and 2, respectively. This process yielded a set of 3,816 unique reads providing a 9.8-fold coverage of the *Bison bison* reference sequence and left 87 positions without coverage. A provisional consensus sequence was then derived from the 3,816 unique selected reads. In this study a consensus for a given position in the genome was based on at least two concordant unique sequences (either Illumina reads or consensus PCR sequences) with a minimal mapping quality of 25 for Illumina reads. The most frequent base was taken as the consensus. The few cases where more than 25% of the bases from the aligned reads differed from the consensus were manually examined: the large majority of the alternative bases could be ascribed to damage-induced G to A substitutions at the 3' end of the Illumina reads and the other cases were checked by PCR sequencing. The Illumina reads aligning to the provisional consensus sequence were retrieved using BWA with the options-*o* 0 and-*n* 0.1, which allows for a maximum edit distance that increases with read length, from one mismatch for 20–26 nucleotide-long reads to four mismatches for 88–101 nucleotide-long reads. This second set of reads contained 3,868 unique reads. Finally PCR experiments were carried out to fill the gaps (39 positions) and derive a robust sequence at positions where a single read was available (176 positions) or where no consensus was reached (5 positions). PCR primers (primer pairs 11–38 in S1 Table, annealing temperature 50 to 55°C) were designed using the provisional genome sequence. The experimental setup for amplification, purification and cloning of the PCR fragments was similar to the one described for the PCR screening procedure of the samples. We performed one to four amplifications with each primer pair and the consensus sequence deduced from an average of 6 clones for each PCR fragment was used to complete the assembly. We noticed a difference between the consensus of all PCRs and the Illumina reads for only 3 out of the 176 positions covered by a single read. The final sequence (SGE2seq) was established by combining the information derived from the Illumina reads and 81 PCR fragments. All the Illumina reads of the library were ultimately aligned to SGE2seq using BWA with the options-*o* 0 and-*n* 0.1, yielding 3,851 unique reads. We verified that the consensus sequence derived from these reads was identical to SGE2seq.

### Sequence annotation

The annotation of SGE2seq was derived from the annotation of the *Bison bison* reference mitochondrial genome. Briefly, SGE2seq and the *Bison bison* reference mitochondrial genome were aligned using the LAGAN software [12] with the annotation data of the *Bison bison* genome (http://www.ncbi.nlm.nih.gov/nuccore/NC_012346.1). LAGAN produced a list of the aligned genomic features with their coordinates in the two genomes, which was manually checked. The occurrence of the expected start and stop codons at the ends of the coding sequences and the absence of internal stop codon were verified using an in-house Python script. The SGE2seq annotated sequence has been deposited in the GenBank database (accession number KM593920).

### Analysis of ancient DNA damage

The mismatches between the 3,851 unique Illumina reads and the SGE2seq sequence were analyzed using in-house Python and R scripts available upon request. Briefly, the SAM files generated by BWA were parsed to retrieve the aligned reads and the corresponding genomic regions, which were compared for mismatches.

### Illumina reads mapping to other *Bos* and *Bison* sequences

To confirm the origin of the SGE2 sample, all the Illumina reads of the library with a minimal length of 30 bp were aligned to the reference mitochondrial genomes of *Bison bison*, *Bison bonasus* (GenBank accession number NC_014044.1 [13]), *Bos primigenius* (GenBank accession number NC_013996.1 [14]) and *Bos taurus* (GenBank accession number NC_006853.1 [15]) using BWA with the options-*o* 0 (no gap opening) and-*n* 0 (perfect match).

To evaluate the coverage of the nuclear genome, the Illumina reads were aligned to the 18S rDNA sequence of *Bos taurus* (extracted from the sequence with GenBank accession number DQ222453.1) using BWA with the options-*o* 0 (no gap opening) and-*n* 1 (one mismatch). The Illumina reads were also aligned to the complete *Bos taurus* nuclear genome generated by inflating and combining the bt_ref_Bos_taurus_UMD_3.1.1_chr*.fa.gz files along with the bt_alt_Btau_4.6.1_chrY.fa.gz file located at ftp://ftp.ncbi.nlm.nih.gov/genomes/Bos_taurus/Assembled_chromosomes/seq/. The mapping was performed using BWA with the options-*o* 0 (no gap opening) and-*n* 0.15.

### Metagenomic analyses

A total of one million reads sampled from the seven sequencing lanes was analyzed by BLAST [16] against the GenBank *nr/nt* database with the following options:-*task* megablast-*word_size* 19-*max_target_seqs* 1-*gapopen* 5-*gapextend* 2. Only the hits that display an E-value lower than 0.01 were considered significant.

### Phylogenetic analyses

Phylogenetic analyses were performed using MEGA [17]. The phylogenetic relationships between the D-loop regions of SGE2seq and of several *Bison priscus* fossils and *Bison bison* specimens were inferred using the Maximum Likelihood method based on the Hasegawa-Kishino-Yano model, as in [2]. The bootstrap consensus tree was built from 1000 replicates. Initial trees for the heuristic search were obtained by applying the Neighbor-Joining method to a matrix of pairwise distances estimated using the Maximum Composite Likelihood approach. The analysis involved a total of 583 nucleotide positions of the D-loop region.

The phylogenetic relationships between the complete mitochondrial genomes of *Bison priscus* (this study), *Bison bison*, *Bison bonasus* and *Bos grunniens* available on the NCBI website (http://www.ncbi.nlm.nih.gov/, last accessed August 4, 2014) along with the reference mitochondrial genomes of *Bos primigenius*, *Bos taurus* and *Bubalus bubalis* (swamp buffalo, GenBank accession number NC_006295.1) were inferred using the Maximum Likelihood, the Minimum Evolution and the Neighbor-Joining methods. The Maximum Likelihood method was based on the General Time Reversible model. A discrete Gamma distribution was used to model evolutionary rate differences among sites (5 categories). The analysis involved a total of 16,315 positions.

### AMS ^14^C dating

Part of the SGE2 sample was broken into 3–4 mm fragments, which were washed in deionized (DI) water with brief sonication to remove adhering sediment. The bone fragments were then decalcified in 0.2 N HCl for 6 hours at 4°C and the acid-insoluble collagen washed with DI water. The collagen was treated with 0.1 M KOH for 2 hours at 4°C, and washed to neutrality with DI water. The alkali-extracted, decalcified collagen was gelatinized in 0.05 N HCl at 90°C for 1 hour and the solution filtered through a 0.45 μm Durapore filter membrane. The soluble collagen was passed through a 30-kDa filter, the > 30 kDa fraction retained and freeze-dried. Purified collagen was then combusted at 850°C in evacuated quartz tubes containing CuO and Ag. The CO_2_ was isolated cryogenically, converted into graphite using the H_2_-Fe method [18] and dated at the W.M. Keck Carbon Cycle Accelerator Mass Spectrometry Laboratory, University of California-Irvine (UCIAMS). The calibrated (cal BP) date range of the sample was obtained using OxCal 4.2 software [19] and the IntCal13 calibration curve [20].

## Results and Discussion

**Fig 1:**
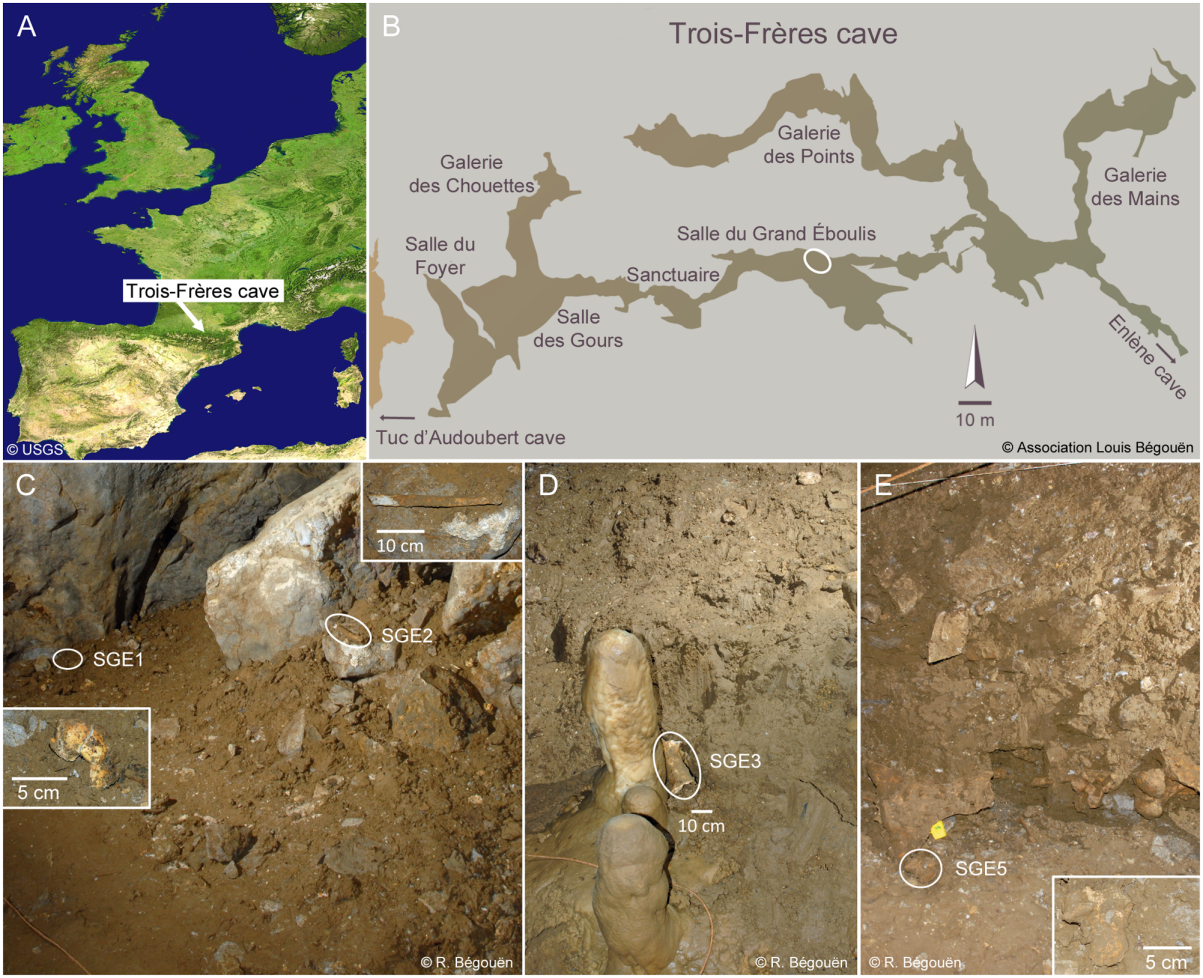
Overview of the cave site and bone samples. (A) Geographic localization of the Trois-Frères cave. (B) Topography of the cave showing the site (white ellipse) where the samples were collected in the *Salle du Grand Éboulis*. (C-E) The samples considered for DNA extraction in the present study. SGE1: ulna distal fragment; SGE2: rib; SGE3: humerus fragment; SGE5, vertebra.

The Trois-Frères cave consists of a series of chambers that spread over 800 meters, from the Enlène cave to the Tuc d’Audoubert cave, from which it is currently disconnected (Fig 1A and 1B). In this cave system the *Salle du Grand Éboulis* (Chamber of the Large Scree) displays unique features. It functioned as a natural trap for Pleistocene animals, and was sealed before the Holocene [21]. The bottom of the scree contains remains of the extinct cave bear (*Ursus spelaeus*), one of which was dated to 36,600–34,800 calBP [21]. In the other layers remains of the extinct steppe bison predominate [22]. They correspond to 19,400 to 17,800-year-old specimens [21].

We analyzed four bone samples that were collected in the *Salle du Grand Éboulis* (SGE) for the purpose of the present study (Fig 1C–1E). Three samples stood on the ground surface, in the vicinity of the pathway delineated since the cave discovery (SGE1, SGE3), or at the bottom of the excavation carried out in the 30’s of the previous century (SGE5). The other sample (SGE2, a rib fragment) stood on a rock, three meters away from the pathway used by modern visitors.

**Fig 2:**
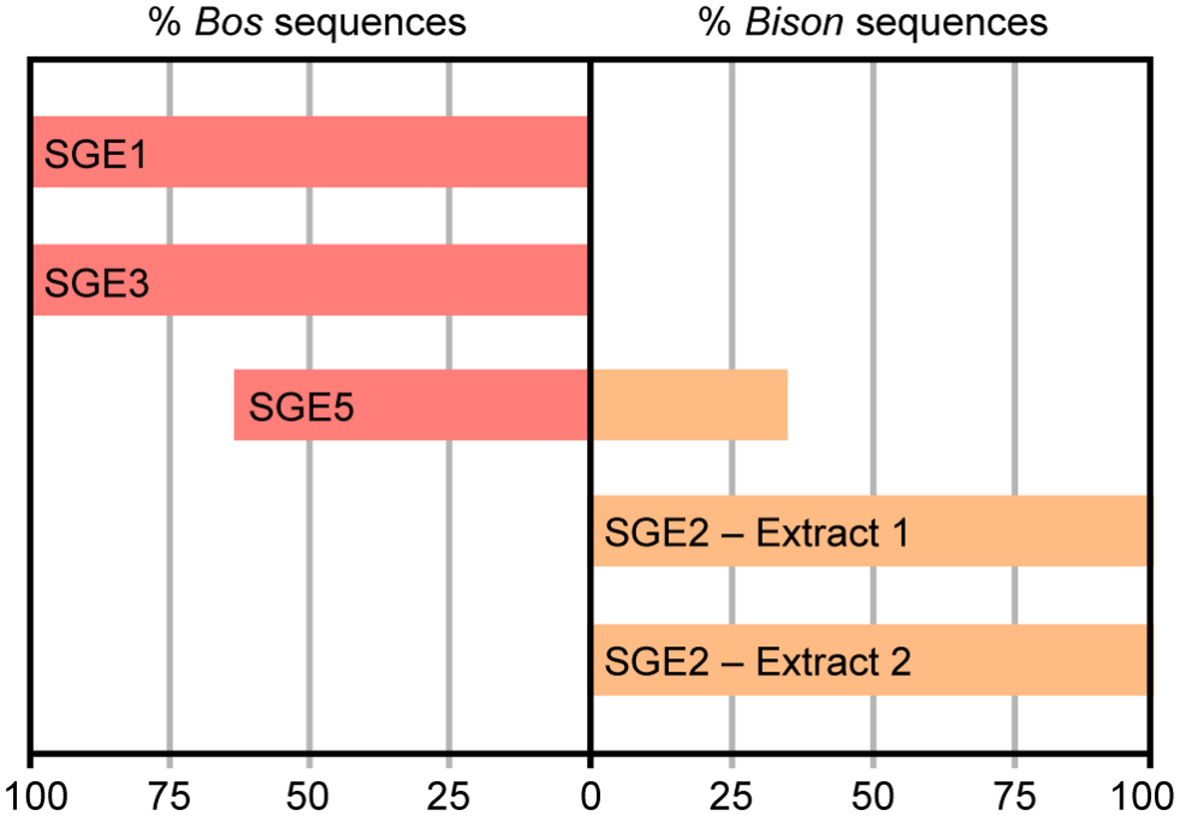
PCR analysis of the samples for *Bovinae* sequences. DNA extracts were analyzed for a fragment of the mitochondrial cytB gene using primers encompassing a 63-bp sequence where *Bos* (*Bos taurus*, *Bos primigenius*) and *Bison* (*Bison bison*, *Bison bonasus*) reference genomes display 5 differences. The cloned PCR products were sequenced and identified as *Bos* or *Bison* according to the number of differences with the reference genomes (see text for details).

We first analyzed the DNA content of the samples by PCR using primer pair 1 (S1 Table). The forward and reverse primers display a perfect match with the *Bos taurus* and *Bos primigenius* reference mitochondrial genome sequences and 0 to 1 mismatch with the *Bison bison* and *Bison bonasus* reference mitochondrial genomes. The mitochondrial DNA sequence flanked by these primers (63-bp) is identical between the *Bos taurus* and *Bos primigenius* reference genomes. *Bison bison* and *Bison bonasus* sequences both display 5 differences (including one transversion) with the two *Bos* genomes, and differ among each other by two transitions. All samples, but none of the negative controls (mock extracts and PCR blanks) yielded amplification products of the predicted size that were characterized through the sequencing of cloned PCR fragments (SGE1: 1 PCR, 13 clones; SGE2 extract 1: 4 PCR, 47 clones; SGE2 extract 2: 2 PCR, 39 clones; SGE3: 1PCR, 12 clones; SGE5: 1PCR, 14 clones). SGE1 and SGE3 DNA extracts yielded sequences that all displayed a perfect match with *Bos* (*Bos taurus*, *Bos primigenius*) reference mitochondrial genomes, and SGE5 returned a mixture of sequences that perfectly matched either *Bos* or *Bison bison* genomes (Fig 2). The PCR data may indicate that SGE1 and SGE3 samples correspond to *Bos* bone fragments, but the observation that SGE5 contains *Bos* as well as *Bison* DNA challenges this interpretation and rather suggests that all samples collected from the ground surface had been contaminated by modern cattle DNA brought by modern human intrusions since the cave discovery. At any rate, none of these three samples could be considered as a relevant candidate for a thorough analysis of bison DNA. The SGE2 bone sample turned out to be much more suitable for this purpose. The two SGE2 DNA extracts yielded sequences that better aligned to *Bison bison* than to *Bos taurus* and *Bos primigenius* mitochondrial genomes (Fig 2 and S2 Table). As a whole, the majority (79%) of these sequences displayed a perfect match with the *Bison bison* reference mitochondrial genome (5 mismatches with *Bos* genomes), the other ones bearing 1–3 and 6–8 differences with the *Bison bison* and *Bos* genomes, respectively.

To further guarantee the relevance of the SGE2 bone sample to study bison DNA, we performed an additional series of PCR experiments using primers designed from *Bison* (primer pairs 2 and 7–10) or *Bos* (primer pairs 3–6) reference mitochondrial genomes. As shown in S2 Table, all these experiments yielded DNA sequences that aligned with higher BLAST scores to *Bison* than to *Bos* mitochondrial genomes. Moreover, using primers targeting the portion of the *Bison priscus* mitochondrial genome made available by previous studies [2], namely the D-loop region, we obtained DNA sequences that clearly exhibited higher alignment scores with this species than with any other one. Thus, the bulk of evidence supported the notion that SGE2 corresponds to a *Bison* bone sample. We therefore decided to further analyze this rib fragment.

**Fig 3:**
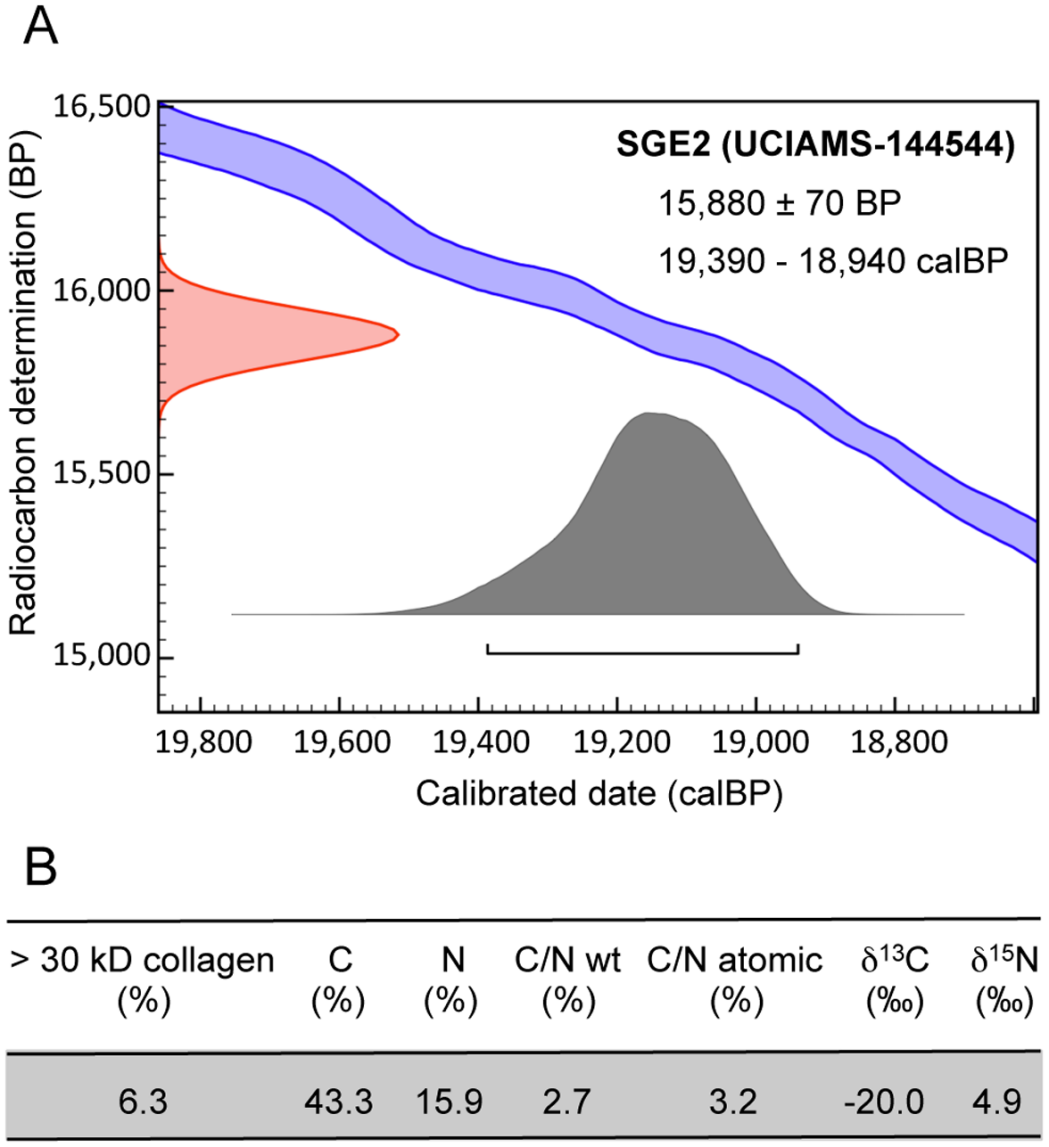
Radiocarbon and stable isotopes data for the SGE2 bison sample. (A) Uncalibrated (B.P.) and calibrated (calBP) age of the bone sample. The calBP data correspond to 95.4% (2 σ) confidence interval of the sample age using IntCal13 calibration curve. (B) Characterization of the dated material.

As shown in Fig 3, SGE2 was radiocarbon dated to 15,880 ± 70 B.P. (19,390–18,940 calBP). It is therefore representative of the age previously obtained for *Bison priscus* bones collected in the *Salle du Grand Éboulis* [21]. In order to obtain genomic data, we produced a library of DNA fragments from the SGE2 sample and performed Illumina sequencing.

A total of 1,033,460,536 Illumina single-pass reads, each at least 20 nucleotides in length, were produced. To confirm the origin of the SGE2 sample and further rule out contamination by modern DNA, the reads were aligned to the reference mitochondrial genomes of *Bison bison*, *Bison bonasus*, *Bos primigenius* and *Bos taurus*. The alignments were performed under stringent conditions: only the reads with a minimal length of 30 bp matching perfectly the target sequence were retained. Under these conditions, 1,825 reads mapped to the *Bison bison* mitochondrial genome. By contrast, only 324, 347 and 331 reads mapped to the mitochondrial genomes of *Bison bonasus*, *Bos primigenius* and *Bos taurus*, respectively. Moreover, the large majority of the reads mapping to the *Bison bonasus* and *Bos* genomes also mapped to the *Bison bison* genome: this was the case for 296 reads out of 324 for the *Bison bonasus* genome, and for 305 reads out of 347 and for 290 reads out of 331, respectively, for the *Bos primigenius* and *Bos taurus* genomes. These results strongly suggest that the reads mapping to the *Bison bonasus* and *Bos* genomes correspond to ancient bison DNA and map to regions that are identical in the genome of *Bison bison* and in the genomes of the other species. The alignment analysis thus corroborated the conclusion, previously drawn from the PCR results, that the SGE2 extract contains *Bison* rather than *Bos* DNA.

To evaluate the coverage of the nuclear genome by the Illumina reads, we analyzed the 18S ribosomal RNA gene, which displays approximately 200 copies in mammalian genomes [23]. Out of ~ 10^9^ reads, 110 aligned to the 18S rDNA sequence of *Bos taurus*. These results correspond to an average read depth of 2.2 for 200 copies and accordingly to ~ 0.01 for single copy sequences. Mapping to the complete nuclear genome of *Bos taurus* yielded 741,848 unique reads with a mapping quality higher than 20. The sum of these reads lengths amounts to 26,117,728 bp, which represents a 0.01-fold coverage of the 2,7 Gb *Bos taurus* nuclear genome, in agreement with our previous estimate. These analyses confirmed the poor coverage of the nuclear genome, which precluded its further analysis.

To gain some further insight into the content of the Illumina library, one million reads were analyzed by BLAST, which yielded 99,684 significant hits. The large majority of these hits (89%) corresponded to *Bacteria* while *Eukaryota* represented only 9% of the total. In the bacterial metagenome, the predominant classes were *Actinobacteria* (32% of all bacterial hits), *Betaproteobacteria* (20%), *Gammaproteobacteria* (18%) and *Alphaproteobacteria* (16%). Similar distributions of microbial diversity have been described for ancient DNA extracts (*e*.*g*. [24]). Among the *Eukaryota*, the family most represented was the *Poaceae* (10% of all eukaryotic hits), followed by the *Bovidae* (6%) and the *Noelaerhabdaceae* (3%). The sum of the lengths of the 540 Illumina reads corresponding to the *Bovidae* hits represents a total of 26,856 nucleotides for one million Illumina reads analyzed, predicting 27,754,600 nucleotides for *Bovidae* sequences in the whole set of reads.

To analyze the *Bison* mitochondrial genome sequence, we first retrieved from the library all the reads that aligned to the mitochondrial genome of *Bison bison* (which, as indicated above, turned out to be more relevant for this purpose than *Bison bonasus*) or to the mitochondrial control region of *Bison priscus* isolate BS212 (GenBank accession number AY748539.1, [2]). This alignment was performed under less stringent conditions than the previous ones and allowed for mismatches (see details in Materials and Methods). A provisional consensus sequence was derived from the selected reads and was used in turn to retrieve all reads aligning to it. PCR experiments were carried out to analyze regions where less than two concordant reads were available. The final sequence, termed SGE2seq, was established using 81 consensus sequences of PCR fragments to complement the information derived from the Illumina reads. All the Illumina reads were aligned one last time to SGE2seq, which yielded 3,851 unique reads.

**Fig 4:**
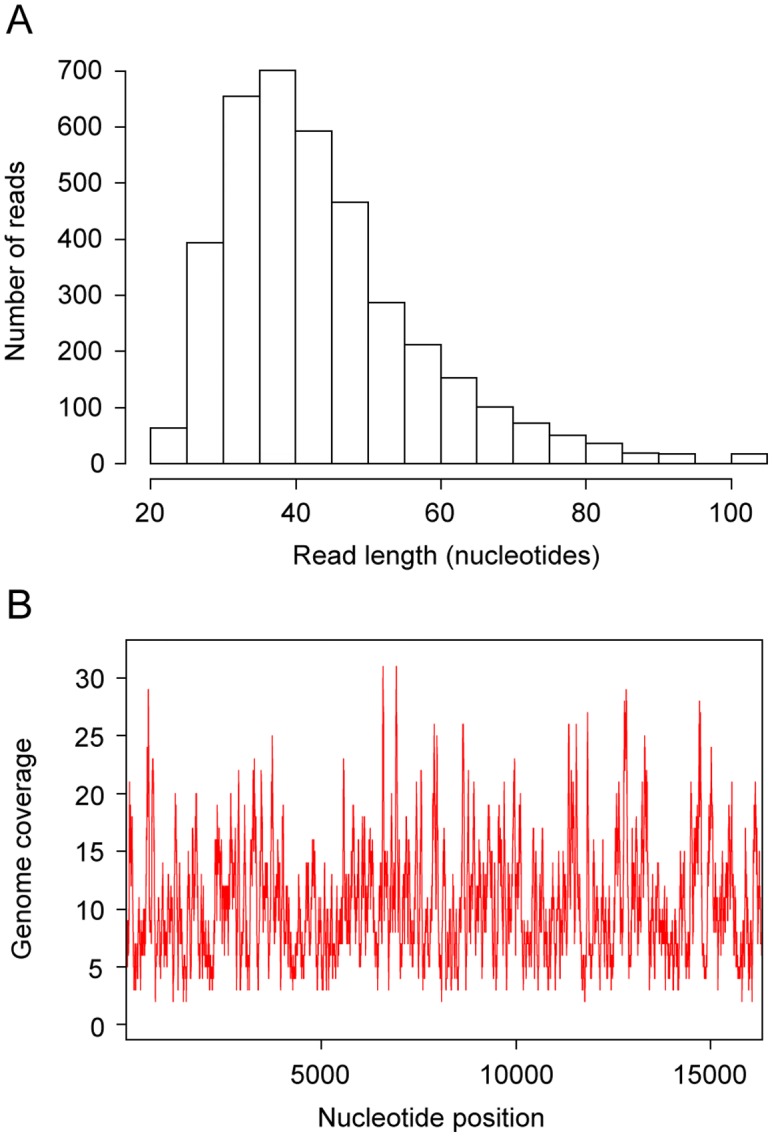
Characteristics of the sequences used to assemble the *Bison priscus* mitochondrial genome. (A) Size distribution of the 3,851 unique Illumina reads. (B) Coverage for each position of the SGE2seq sequence obtained by merging the sequences of Illumina reads and PCR fragments.

SGE2seq consists of a circular genome, for which we obtained a 10.4-fold coverage, taking into account the 3,851 unique Illumina reads and the 81 PCR sequences. Fig 4A shows the length distribution of the 3,851 unique Illumina reads and Fig 4B, the coverage obtained for each position.

Only 1,443 mismatches out of 166,849 aligned bases are observed between the 3,851 unique Illumina reads and the consensus sequence SGE2seq, which corresponds to 99.1% identity. As shown in S1 Fig, the G to A substitution rate increases at 3’ ends, in agreement with what has already been reported for libraries of ancient DNA constructed with the same procedure [25]. An increased substitution rate at DNA ends is considered as a hallmark authenticating sequences generated from ancient DNA fragments which exhibit inflated cytosine deamination rates at 5'-overhangs [26], responsible for G to A transitions at the 3’ end of the opposite, repaired DNA strand.

SGE2seq corresponds to a mitochondrial genome sequence of 16,318 bp. This length is similar to the lengths of *Bison bison* mitochondrial genomes, which range from 16,318 to 16,323 bp [27]. The genome consists of 13 protein-coding genes, 22 tDNA genes, 2 rDNA genes, and the D-loop region (S3 Table).

Since part of the D-loop region has been sequenced for a number of *Bison priscus* fossils [2,4], we analyzed the phylogenetic relationships between the orthologous portion of SGE2seq and of several *Bison priscus* fossils and *Bison bison* specimens. We also considered the D-loop region of several reference genomes of *Bison* and *Bos* species, including the *Bos grunniens* (yak) reference mitochondrial genome (GenBank accession number NC_006380). The phylogenetic reconstruction, carried out using the Maximum Likelihood method, positions SGE2seq among *Bison priscus* fossils sequences, outside the strongly supported clade of modern *Bison bison* sequences (S2 Fig). This result was observed when taking into account three specimens from each of the four *Bison* groups defined in [2], and similar results were obtained when including all published D-loop sequences of *Bison priscus* and *Bison bison* (data not shown). The position of SGE2seq D-loop region in the phylogenetic tree further corroborates the assumption that SGE2seq corresponds to the mitochondrial genome of a *Bison priscus* specimen.

Several lines of evidence indicate that we obtained a reliable mitochondrial genome sequence for a Pleistocene *Bison priscus* specimen. First, the DNA material was retrieved from a 19,390 to 18,940-year-old bone sample (Fig 3). Second, considering the mitochondrial genome portion (600 bp of the D-loop) previously characterized for the extinct steppe bison [2,4], phylogenetic analysis demonstrated that the sequence assembled from our sample positions in the cluster delineated by *Bison priscus* specimens (S2 Fig). Third, we observed the damage-induced misincorporation pattern expected for ancient DNA (S1 Fig). Fourth, although of limited magnitude, the average unique read depth (10.4-fold) is large enough to overcome the error rate (< 1%, as judged by comparing the reads with the consensus sequence), and positions poorly covered were checked by PCR and sequencing of multiple cloned amplicons. Fifth, we obtained a circular genome that contains the expected number of genes (rRNAs, tRNAS, protein-coding genes) for a mammalian mitochondrial genome, with full-length coding sequences (without internal stop codons) for all 13 protein-coding genes. Thus, it is safe to conclude that we provide an authentic and complete *Bison priscus* mitochondrial genome sequence.

When single-nucleotide polymorphisms (SNPs) and insertion-deletion events are considered, there are 106–117 differences between SGE2seq and the 33 published *Bison bison* mitochondrial genomes (*Bison bison* genomes differ among themselves at 24 positions at most). SGE2seq shows 114 differences (listed in S4 Table) compared to the reference *Bison bison* mitochondrial genome, including two indels: a 1-bp insertion located in the D-loop region and a 2-bp deletion at the end of the tRNA serine gene (this shorter tRNA serine allele is also present in some *Bison bison* mitochondrial genomes [27]). The other differences comprise 105 transitions and 6 transversions. A similar, strong transitional bias for mitochondrial genomes has also been reported for *Bos* species [14]. Twenty-five differences (22%) are located in the D-loop region, which therefore exhibits a mutation rate four times higher than the rest of the genome. Finally, 73 substitutions (65%) are located in protein-coding genes, with 16, 3 and 54 substitutions occurring in the first, second and third codon positions, respectively. These substitutions correspond to 11 amino acid differences. Among the 114 differences, 93 correspond to polymorphisms that are specific to *Bison priscus* and are not found in any published *Bison bison* genome (S5 Table). These 93 specific polymorphisms are mostly located in protein-coding genes (69%) and correspond to 7 amino acids differences.

**Fig 5:**
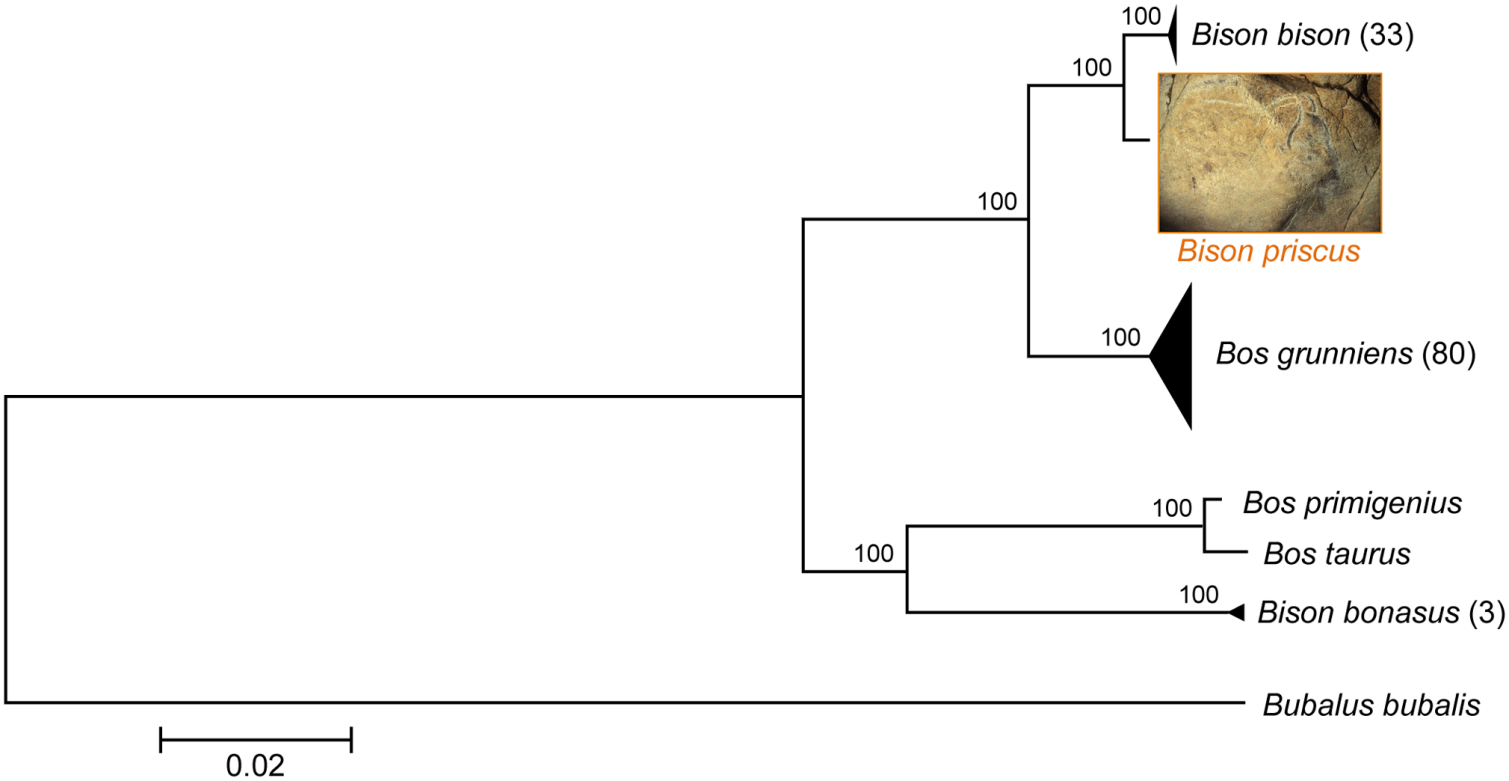
Maximum Likelihood phylogenetic tree of complete mitochondrial genomes of *Bison* and *Bos* species. The numbers indicated in parentheses after the species name refer to the number of genomes examined for that species (which is equal to 1 by default). The tree with the highest log-likelihood is shown drawn to scale, with branch lengths established from the number of substitutions per site. The same topology was obtained with the Minimum Evolution (ME) and Neighbor-Joining (NJ) methods. The percentages of trees in which the associated taxa clustered together with the ML method are displayed next to the branches (these bootstrap values were determined with 1000 replicates). These percentages were also equal to 100 with the ME and the NJ methods. Photography of painted and engraved bison by Robert Bégouën.

A phylogenetic analysis was performed using all the available complete mitochondrial genomes of *Bison bison*, *Bison bonasus* and *Bos grunniens* along with the reference mitochondrial genomes of *Bos primigenius*, *Bos taurus* and *Bubalus bubalis* (swamp buffalo). Phylogenetic trees were constructed from this dataset with the Maximum Likelihood (ML), Minimum Evolution (ME) and Neighbor-Joining (NJ) methods. As shown in Fig 5 for the ML method, SGE2seq is basal to the modern *Bison bison* clade, and the set including SGE2seq and all *Bison bison* sequences forms a distinct and well-supported (100% bootstrap support, 1000 replicates) clade. Similar results were obtained with the ME and NJ methods, which conclusively supports the contention that SGE2seq corresponds to a *bona fide* mitochondrial sequence of *Bison priscus*.

In summary, we have presented here the analysis of a 19,000-year-old bone specimen that made it possible to obtain the first complete mitochondrial genome for the extinct steppe bison. Phylogenetic reconstruction unequivocally points to the basal position of the *Bison priscus* mitochondrial genome as compared to the clade delineated by extant *Bison bison* genomes. The sequence provided here may be a useful tool for the genomic analysis of other ancient *Bovinae* specimens. Our study also shows that the Trois-Frères cave system, already famous for Magdalenian art, evidence of human habitation and abundant animal remains, can now be considered for paleogenetic and paleogenomic analyses.

## Supporting Information

S1 Fig Position of the mismatches between the 3,851 unique Illumina reads and the SGE2seq sequence. The frequencies of the 12 possible mismatches are plotted as a function of the distance from the 5' end (A) or the 3' end (B) of reads. Since the Illumina reads are at least 20 nucleotides in length, only the ten 5' and ten 3' most positions are shown.
(TIF)

S2 Fig Maximum Likelihood phylogenetic tree of the D-loop sequences of *Bison* and *Bos* species. Branches corresponding to partitions reproduced in less than 50% bootstrap replicates are collapsed. The percentage of replicate trees in which the associated taxa clustered together in the bootstrap test is shown next to the branches. The D-loop fragment of SGE2seq is boxed in red (*Bison priscus* TF for "Trois-Frères") and the D-loop sequences of the *Bison bison* clade are boxed in blue.
(TIF)

S1 Table PCR primers used in this study. Primer pair 1 was used to analyze all four bone samples for *Bos* and *Bison* mitochondrial DNA. Primer pairs 2–10 were used to characterize further the SGE2 sample before initiating Illumina sequencing. Primer pairs 11–38 were used to fill in the gaps in the genome reconstructed by shotgun DNA sequencing and check the sequence of the genome regions where a single Illumina read was obtained. The position of each forward (F) and reverse (R) primer is numbered according to the SGE2seq sequence.
(DOCX)

S2 Table PCR analysis of the SGE2 bone sample. Each line provides the results for a PCR sample obtained with one primer pair (column 1) and characterized through the sequencing of the indicated number of cloned fragments (column 2, number of sequences). Columns 3 and 4 indicate the size of the sequence located between the primers and its location in the mitochondrial genome, respectively. The consensus deduced from the sequence of the cloned amplicons was analyzed by BLAST against the GenBank *nr/nt* database. Results of BLAST analysis for the indicated species are displayed in columns 5–19. NA: not applicable (the corresponding genome portion of *Bison priscus* was not available in GenBank).
(DOCX)

S3 Table Annotation of the *Bison priscus* mitochondrial genome. The intergenic nucleotides column indicates the number of intergenic or overlapping nucleotides ("-n" indicates overlapping regions).
(DOCX)

S4 Table List of the differences between the mitochondrial genome sequence of *Bison priscus* and the reference genome sequence of *Bison bison*. The Position column indicates the position of the difference with respect to the *Bison priscus* sequence; the Strand column, the strand of the feature (irrelevant for intergenic regions); the Position codon column, the position of the polymorphism within the codon (for coding sequences); the Base priscus and the Base bison columns, the polymorphic bases for the *Bison priscus* and *Bison bison* sequences, respectively; the Sequence priscus and the Sequence bison columns, the 5-bp sequences where the polymorphism is located (the polymorphic base is in the middle); and the Type column, the type of difference between the *Bison priscus* and the *Bison bison* sequences ("t" for transition, "v" for transversion). NA, not applicable.
(DOCX)

S5 Table List of the polymorphisms that are specific to the genome of *Bison priscus*. The compendium was established by comparing SGE2seq to the genome sequences available for 33 modern *Bison bison* specimens. The column names are as in S4 Table.
(DOCX)
