# Supplementary figures and images for: Hunting the Extinct Steppe Bison (Bison priscus) Mitochondrial Genome in the Trois-Frères Paleolithic Painted Cave
Source: PLoS One. 2015 Jun 17;10(6):e0128267. doi: 10.1371/journal.pone.0128267 (PMC4471230; doi:10.1371/journal.pone.0128267)

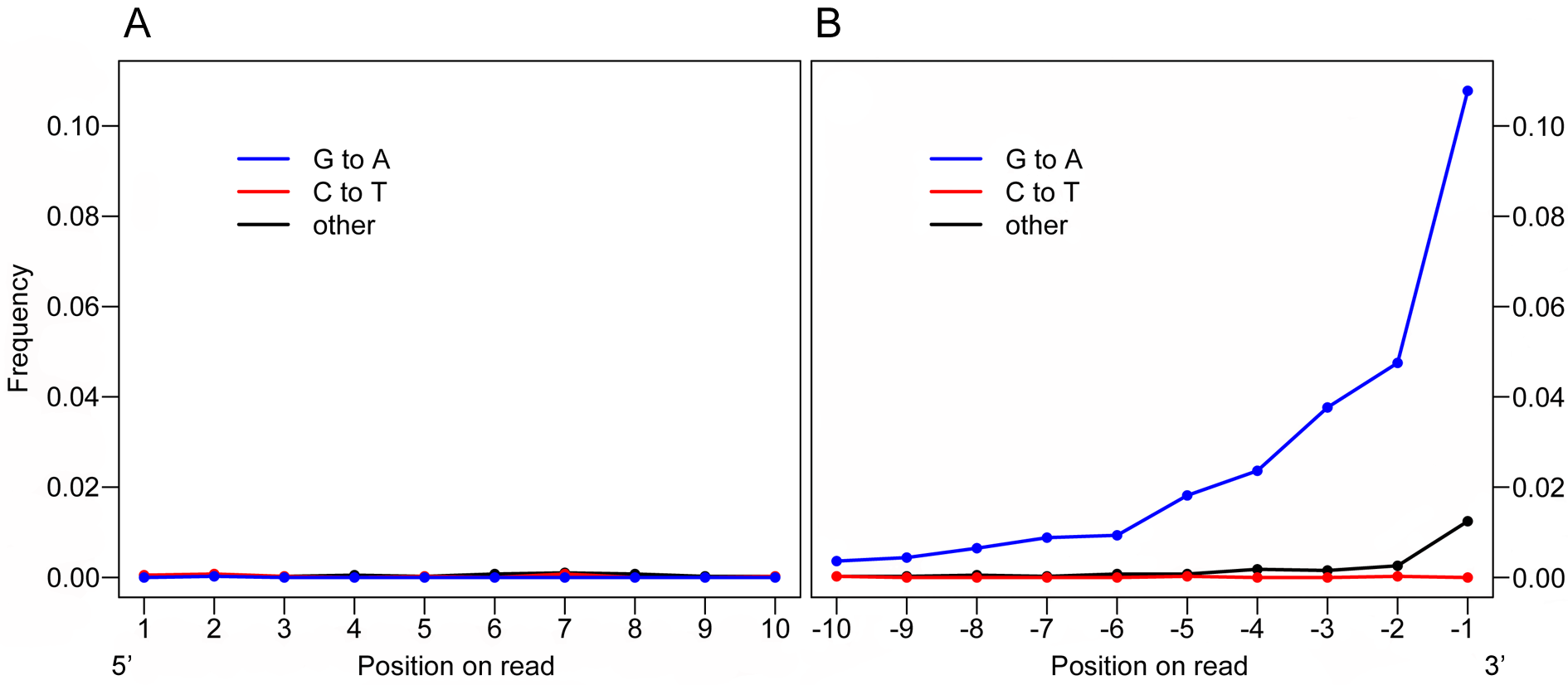

Supplement: S1 Fig — The frequencies of the 12 possible mismatches are plotted as a function of the distance from the 5' end (A) or the 3' end (B) of reads. Since the Illumina reads are at least 20 nucleotides in length, only the ten 5' and ten 3' most positions are shown. (TIF) [file pone.0128267.s001.tif]

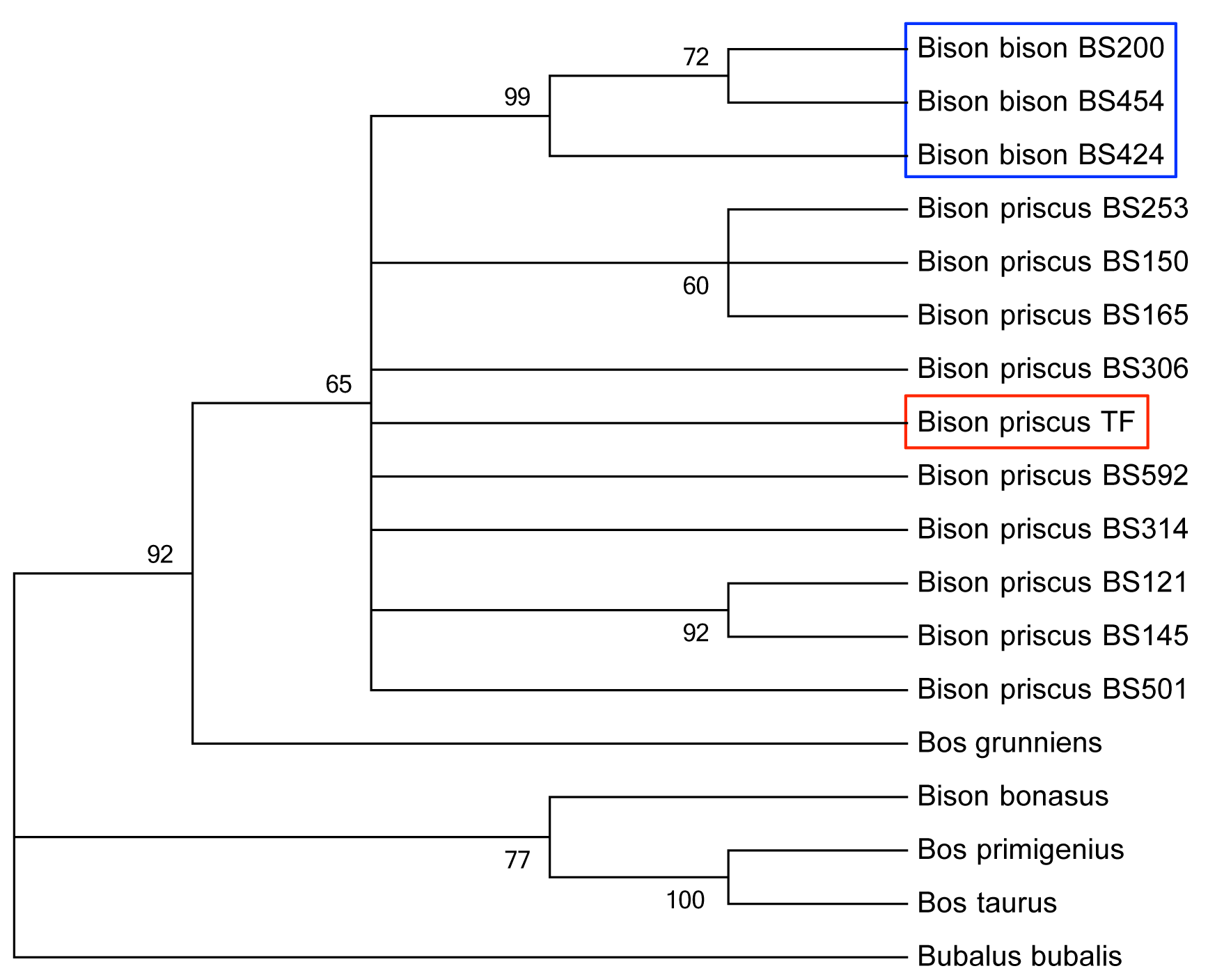

Supplement: S2 Fig — Branches corresponding to partitions reproduced in less than 50% bootstrap replicates are collapsed. The percentage of replicate trees in which the associated taxa clustered together in the bootstrap test is shown next to the branches. The D-loop fragment of SGE2seq is boxed in red (Bison priscus TF for "Trois-Frères") and the D-loop sequences of the Bison bison clade are boxed in blue. (TIF) [file pone.0128267.s002.tif]
