# Supplementary material for: Hunting the Extinct Steppe Bison (Bison priscus) Mitochondrial Genome in the Trois-Frères Paleolithic Painted Cave
Source: PLoS One. 2015 Jun 17;10(6):e0128267. doi: 10.1371/journal.pone.0128267 (PMC4471230; doi:10.1371/journal.pone.0128267)
Supplement: S1 Table — Primer pair 1 was used to analyze all four bone samples for Bos and Bison mitochondrial DNA. Primer pairs 2–10 were used to characterize further the SGE2 sample before initiating Illumina sequencing. Primer pairs 11–38 were used to fill in the gaps in the genome reconstructed by shotgun DNA sequencing and check the sequence of the genome regions where a single Illumina read was obtained. The position of each forward (F) and reverse (R) primer is numbered according to the SGE2seq sequence. (DOCX) [file pone.0128267.s003.docx]

**S1 Table.** PCR primers used in this study

| Primer pair | Primer position, sequence | Amplicon (bp) |
| --- | --- | --- |
| 1 | 15259F, 5’-CCTCGGAGACCCAGATAACT-3’  15362R, 5’-TTGATCGTAAGATTGCGTATG-3’ | 104 |
| 2 | 10052F, 5’-GAGCCTCACAAACAACAAACTT-3’  10175R, 5’-GTACCATATTCGGTTCATTCTAGTC-3’ | 124 |
| 3 | 14711F, 5’-ACCCATATCTGCCGAGAC-3’  14812R, 5’-TCGTCCTACGTGCATATATAA-3’ | 102 |
| 4 | 14768F, 5’-GGAGCTTCAATGTTTTTTATCTG-3’  14867R, 5’-GGATTACTCCAATATTTCATGTTT-3’ | 100 |
| 5 | 14818F, 5’-ATATTACGGGTCTTACACTTTTC-3’  14919R, 5’-CCTCATGGTAGGACGTATCC-3’ | 102 |
| 6 | 14936F, 5’-GGAGCAACAGTCATCACCAA-3’  15053R, 5’-AAGCGAAGAATCGGGTAAGG-3’ | 118 |
| 7 | 15357F, 5’-GATCAATCCCCAACAAAC-3’  15438R, 5’-GTGTGTAGTAGGGGAATTAGA-3’ | 82 |
| 8 | 15883F, 5’-AACCAAATATTACAAACACCACTA-3’  16012R, 5’-GGGGCATATAATTTAATGTACT-3’ | 130 |
| 9 | 16010F, 5’-CCCATGCATATAAGCAAGT-3’  16148R, 5’-TGGTAATTAAGCTCGTGATCTA-3’ | 139 |
| 10 | 16162F, 5’-CAGCAACCCGCTAGGCA-3’  16266R, 5’-GCCCTGAAGAAAGAACCAGATGT-3’ | 105 |
| 11 | 156F, 5’-CATAATGGTAGGCATGCACA-3’  254R, 5’-AGTGGTAAATATTTAAGGGGGAA-3’ | 99 |
| 12 | 156F, 5’-CATAATGGTAGGCATGCACA-3’  270R, 5’-AAAATCTGTTAAAAGCAGTGGT-3’ | 115 |
| 13 | 304F, 5’-CAATACTCAAATTAGCACTCCA-3’  399R, 5’-ATTTTCAGTGCCTTGCTTT-3’ | 96 |
| 14 | 1394F, 5’-AGCTTAAACAAAGCATCCAGT-3’  1483R, 5’-AGTTAAGAGGGTATTTTGGGCTA-3’ | 90 |
| 15 | 1457F, 5’-AATCTAGCCCAAAATACCCTC-3’  1559R, 5’-CGCCATATTCTAGATTTCTATCTCC-3’ | 103 |
| 16 | 1900F, 5’-GGTACAGCCTTTTAGAAACGGAT-3’  1998L, 5’-TTAACGCTTTCTCAATTGGTGG-3’ | 99 |
| 17 | 2013F, 5’-AATTAAACAGATCCCAATAACA-3’  2132R, 5’-CTTATGCAAGGAGAAAGTT-3’ | 120 |
| 18 | 3060F, 5’-AACTTTTATACCCAGAGATTCA-3’  3177R, 5’-TTTTCGTTCTACTAATGTGAGG-3’ | 118 |
| 19 | 4024F, 5’-ATCCTTACATCAGGCATCCCA-3’  4134R, 5’-CCTATAGTTCTAGAAATAAGAGGGTT-3’ | 111 |
| 20 | 4037F, 5’-GCATCCCACCACAAACAT-3’  4156R, 5’-ATTCTTAGGAGTAGGTTCAATTC-3’ | 120 |
| 21 | 4251F, 5’-CCCTTCCCGTACTAATAAATCCAA-3’  4359R, 5’-CCGATTCAGACAAGTAGTCAGT-3’ | 109 |
| 22 | 4439F, 5’-TATTTTCTAACCCAATCAACAGC-3’  4539R, 5’-GGGTTAAATAATTTTATTACGGTTCA-3’ | 101 |
| 23 | 5179F, 5’-CCCCTCTACAAACAACAT-3’  5258R, 5’-ATACGGCTATTGTTGGTAGG-3’ | 80 |
| 24 | 5493F, 5’-ACCCCAATTAATCAGGCTTC-3’  5605R, 5’-AGTTTACACGTTGAATTGCAAA-3’ | 113 |
| 25 | 6431F, 5’-CCTGGATTTGGAATAATCTCCC-3’  6513R, 5’-GCTCAGACTATTCCTATATATCCG-3’ | 83 |
| 26 | 7264F, 5’-TTCAAGCCAACATCATAACCTC-3’  7357R, 5’-TTTCACTTGTAACTTAACTTTGACA-3’ | 94 |
| 27 | 8219F, 5’-CACAACTTTCACTACAATCCAG-3’  8301R, 5’-ATTTTCGTTCATTTTGTTTCTCA-3’ | 83 |
| 28 | 8290F, 5’-ATGAACGAAAATTTATTTGCCTCT-3’  8403R, 5’-CACTAATCGATTTGATGTTGGGA-3’ | 114 |
| 29 | 11068F, 5’-ATACTGAGTACAACCTGTGCAT-3’  11173R, 5’-TCAAAGGTGGAGGCCGTAT-3’ | 106 |
| 30 | 11838F, 5’-TATCGCTGCACATCTTACCT-3’  11939R, 5’-CACAATCTAATGTTTTTGTTAAACT-3’ | 102 |
| 31 | 11851F, 5’-CTTACCTTTACTACTCTTATCCCT-3’  11960R, 5’-ATGAGTTTCTATTGTTAGATTCAC-3’ | 110 |
| 32 | 12077F, 5’-CGAAAAATTGGTGCAACTCC-3’  12178R, 5’-AGGCTTGTTATTATAATGGGTAC-3’ | 102 |
| 33 | 12320F, 5’-CCAAACCCTTAAACTATCCCTC-3’  12393R, 5’-CGAATAGTGCTACTGGGACA-3’ | 74 |
| 34 | 12876F, 5’-ATCTTCCTATTAATCCGCTTT-3’  12977R, 5’-TATCGCTGTAAATAACGTGGT-3’ | 102 |
| 35 | 14087F, 5’-TCAAACACAATCTCTACCTCC-3’  14173R, 5’-TTTAGGGGCATTTATTACTGG-3’ | 87 |
| 36 | 14353F, 5’-CCCATAAATAGGTGAAGGC-3’  14457R, 5’-AGATTCCATGTAAGAATAATGA-3’ | 105 |
| 37 | 15547F, 5’-CCCATATATCATCATCGGACA-3’  15639R, 5’-AGTTTGTTTTCAATTGTGCC-3’ | 93 |
| 38 | 15922F, 5’-CCCCAAAATGCATTACCCAA-3’  16016R, 5’-GCATGGGGCATATAATTTAATGT-3’ | 95 |

Primer pair 1 was used to analyze all four bone samples for *Bos* and *Bison* mitochondrial DNA. Primer pairs 2-10 were used to characterize further the SGE2 sample before initiating Illumina sequencing. Primer pairs 11-38 were used to fill in the gaps in the genome reconstructed by shotgun DNA sequencing and check the sequence of the genome regions where a single Illumina read was obtained. The position of each forward (F) and reverse (R) primer is numbered according to the SGE2seq sequence.
