# Supplementary material for: Hunting the Extinct Steppe Bison (Bison priscus) Mitochondrial Genome in the Trois-Frères Paleolithic Painted Cave
Source: PLoS One. 2015 Jun 17;10(6):e0128267. doi: 10.1371/journal.pone.0128267 (PMC4471230; doi:10.1371/journal.pone.0128267)
Supplement: S2 Table — Each line provides the results for a PCR sample obtained with one primer pair (column 1) and characterized through the sequencing of the indicated number of cloned fragments (column 2, number of sequences). Columns 3 and 4 indicate the size of the sequence located between the primers and its location in the mitochondrial genome, respectively. The consensus deduced from the sequence of the cloned amplicons was analyzed by BLAST against the GenBank nr/nt database. Results of BLAST analysis for the indicated species are displayed in columns 5–19. NA: not applicable (the corresponding genome portion of Bison priscus was not available in GenBank). (DOCX) [file pone.0128267.s004.docx]

**S2 Table.** PCR analysis of the SGE2 sample

| Primer  pair | Number of sequences | Query  (bp) | Genome  portion | *Bos taurus* | | | *Bos primigenius* | | | *Bison bonasus* | | | *Bison bison* | | | *Bison priscus* | | |
| --- | --- | --- | --- | --- | --- | --- | --- | --- | --- | --- | --- | --- | --- | --- | --- | --- | --- | --- |
|  |  |  |  | Query  cover (%) | E value | Identity  (%) | Query  cover (%) | E value | Identity  (%) | Query  cover (%) | E value | Identity  (%) | Query  cover (%) | E value | Identity  (%) | Query  cover (%) | E value | Identity  (%) |
| 1 | 28 | 63 | CYTB | 100% | 2E-17 | 94% | 100% | 8E-16 | 92% | 100% | 4E-20 | 97% | 100% | 7E-23 | 100% | NA | NA | NA |
| 1 | 8 | 63 | CYTB | 100% | 2E-17 | 94% | 100% | 8E-16 | 92% | 100% | 4E-20 | 97% | 100% | 7E-23 | 100% | NA | NA | NA |
| 1 | 3 | 63 | CYTB | 100% | 2E-17 | 94% | 100% | 8E-16 | 92% | 100% | 4E-20 | 97% | 100% | 7E-23 | 100% | NA | NA | NA |
| 1 | 8 | 63 | CYTB | 100% | 2E-17 | 94% | 100% | 8E-16 | 92% | 100% | 4E-20 | 97% | 100% | 7E-23 | 100% | NA | NA | NA |
| 1 | 33 | 63 | CYTB | 100% | 2E-17 | 94% | 100% | 8E-16 | 92% | 100% | 4E-20 | 97% | 100% | 7E-23 | 100% | NA | NA | NA |
| 1 | 6 | 63 | CYTB | 100% | 8E-16 | 90% | 100% | 3E-14 | 89% | 100% | 2E-18 | 94% | 100% | 3E-21 | 97% | NA | NA | NA |
| 2 | 7 | 77 | ND3 | 100% | 1E-27 | 97% | 100% | 5E-26 | 96% | 100% | 3E-23 | 94% | 100% | 1E-28 | 99% | NA | NA | NA |
| 2 | 6 | 77 | ND3 | 100% | 5E-26 | 96% | 100% | 7E-25 | 95% | 100% | 3E-22 | 92% | 100% | 1E-27 | 97% | NA | NA | NA |
| 3 | 8 | 63 | CYTB | 100% | 4E-20 | 97% | 100% | 4E-20 | 97% | 100% | 2E-21 | 92% | 100% | 7E-23 | 100% | NA | NA | NA |
| 3 | 6 | 63 | CYTB | 100% | 4E-20 | 97% | 100% | 4E-20 | 97% | 100% | 2E-21 | 92% | 100% | 7E-23 | 100% | NA | NA | NA |
| 3 | 8 | 63 | CYTB | 100% | 4E-20 | 97% | 100% | 4E-20 | 97% | 100% | 2E-21 | 92% | 100% | 7E-23 | 100% | NA | NA | NA |
| 4 | 7 | 53 | CYTB | 100% | 3E-13 | 94% | 100% | 3E-13 | 94% | 100% | 3E-13 | 94% | 100% | 5E-16 | 98% | NA | NA | NA |
| 4 | 8 | 53 | CYTB | 100% | 3E-12 | 92% | 100% | 3E-12 | 92% | 100% | 3E-12 | 92% | 100% | 1E-17 | 100% | NA | NA | NA |
| 4 | 8 | 53 | CYTB | 100% | 3E-12 | 92% | 100% | 3E-12 | 92% | 100% | 3E-12 | 92% | 100% | 1E-17 | 100% | NA | NA | NA |
| 4 | 8 | 53 | CYTB | 100% | 3E-12 | 92% | 100% | 3E-12 | 92% | 100% | 3E-12 | 92% | 100% | 1E-17 | 100% | NA | NA | NA |
| 5 | 15 | 59 | CYTB | 100% | 2E-15 | 93% | 100% | 2E-15 | 93% | 100% | 1E-13 | 92% | 100% | 4E-19 | 98% | NA | NA | NA |
| 6 | 6 | 78 | CYTB | 100% | 2E-25 | 95% | 100% | 2E-25 | 95% | 100% | 2E-25 | 95% | 100% | 4E-28 | 97% | NA | NA | NA |
| 6 | 4 | 78 | CYTB | 100% | 2E-26 | 96% | 100% | 2E-26 | 96% | 100% | 2E-26 | 96% | 100% | 3E-29 | 99% | NA | NA | NA |
| 6 | 6 | 78 | CYTB | 100% | 2E-26 | 96% | 100% | 2E-26 | 96% | 100% | 2E-26 | 96% | 100% | 3E-29 | 99% | NA | NA | NA |
| 6 | 8 | 78 | CYTB | 100% | 2E-26 | 96% | 100% | 2E-26 | 96% | 100% | 2E-26 | 96% | 100% | 3E-29 | 99% | NA | NA | NA |
| 6 | 7 | 78 | CYTB | 100% | 2E-25 | 95% | 100% | 2E-25 | 95% | 100% | 2E-25 | 95% | 100% | 4E-28 | 97% | NA | NA | NA |
| 7 | 16 | 43 | CYTB | 100% | 7E-12 | 98% | 100% | 7E-12 | 98% | 93% | 5E-10 | 98% | 100% | 3E-14 | 100% | NA | NA | NA |
| 8 | 16 | 84 | D-loop | 100% | 2E-19 | 88% | 82% | 3E-11 | 85% | 82% | 1E-09 | 83% | 100% | 2E-31 | 98% | 100% | 2E-32 | 99% |
| 9 | 8 | 98 | D-loop | 90% | 8E-26 | 91% | 88% | 5E-22 | 89% | 89% | 4E-20 | 83% | 100% | 2E-33 | 94% | 100% | 6E-40 | 99% |
| 9 | 8 | 98 | D-loop | 90% | 2E-26 | 90% | 88% | 2E-20 | 87% | 89% | 4E-17 | 84% | 100% | 9E-32 | 93% | 100% | 7E-39 | 98% |
| 10 | 8 | 65 | D-loop | 98% | 9E-22 | 98% | 98% | 6E-18 | 94% | 98% | 6E-18 | 94% | 100% | 2E-18 | 94% | 100% | 6E-24 | 100% |
| 10 | 8 | 65 | D-loop | 98% | 9E-22 | 98% | 98% | 6E-18 | 94% | 98% | 6E-18 | 94% | 100% | 2E-18 | 94% | 100% | 6E-24 | 100% |
| 10 | 8 | 65 | D-loop | 98% | 9E-22 | 98% | 98% | 6E-18 | 94% | 98% | 6E-18 | 94% | 100% | 2E-18 | 94% | 100% | 6E-24 | 100% |

Each line provides the results for a PCR sample obtained with one primer pair (column 1) and characterized through the sequencing of the indicated number of cloned fragments (column 2, number of sequences). Columns 3 and 4 indicate the size of the sequence located between the primers and its location in the mitochondrial genome, respectively. The consensus deduced from the sequence of the cloned amplicons was analyzed by BLAST against the GenBank *nr/nt* database. Results of BLAST analysis for the indicated species are displayed in columns 5-19. NA: not applicable (the corresponding genome portion of *Bison priscus* was not available in GenBank).
