# Supplementary material for: Hunting the Extinct Steppe Bison (Bison priscus) Mitochondrial Genome in the Trois-Frères Paleolithic Painted Cave
Source: PLoS One. 2015 Jun 17;10(6):e0128267. doi: 10.1371/journal.pone.0128267 (PMC4471230; doi:10.1371/journal.pone.0128267)
Supplement: S3 Table — The intergenic nucleotides column indicates the number of intergenic or overlapping nucleotides ("-n" indicates overlapping regions). (DOCX) [file pone.0128267.s005.docx]

**S3 Table.** Annotation of the *Bison priscus* mitochondrial genome sequence.

| Feature | Strand | Start | Stop | Length | Intergenic nucleotides |
| --- | --- | --- | --- | --- | --- |
| D-loop | + | 1 | 361 | 361 | 1 |
| tRNA-Phe | + | 363 | 429 | 67 | 0 |
| s-rRNA | + | 430 | 1385 | 956 | 0 |
| tRNA-Val | + | 1386 | 1452 | 67 | 0 |
| l-rRNA | + | 1453 | 3022 | 1570 | 0 |
| tRNA-Leu | + | 3023 | 3097 | 75 | 2 |
| ND1 | + | 3100 | 4055 | 956 | 0 |
| tRNA-Ile | + | 4056 | 4124 | 69 | -3 |
| tRNA-Gln | - | 4122 | 4193 | 72 | 2 |
| tRNA-Met | + | 4196 | 4264 | 69 | 0 |
| ND2 | + | 4265 | 5306 | 1042 | 0 |
| tRNA-Trp | + | 5307 | 5373 | 67 | 1 |
| tRNA-Ala | - | 5375 | 5443 | 69 | 1 |
| tRNA-Asn | - | 5445 | 5518 | 74 | 0 |
| rep_origin | + | 5519 | 5549 | 31 | 1 |
| tRNA-Cys | - | 5551 | 5617 | 67 | 0 |
| tRNA-Tyr | - | 5618 | 5685 | 68 | 1 |
| COX1 | + | 5687 | 7231 | 1545 | -3 |
| tRNA-Ser | - | 7229 | 7299 | 71 | 4 |
| tRNA-Asp | + | 7304 | 7372 | 69 | 1 |
| COX2 | + | 7374 | 8057 | 684 | 3 |
| tRNA-Lys | + | 8061 | 8127 | 67 | 1 |
| ATP8 | + | 8129 | 8329 | 201 | -40 |
| ATP6 | + | 8290 | 8970 | 681 | -1 |
| COX3 | + | 8970 | 9750 | 781 | 3 |
| tRNA-Gly | + | 9754 | 9822 | 69 | 0 |
| ND3 | + | 9823 | 10169 | 347 | 0 |
| tRNA-Arg | + | 10170 | 10238 | 69 | 0 |
| ND4L | + | 10239 | 10535 | 297 | -7 |
| ND4 | + | 10529 | 11906 | 1378 | 0 |
| tRNA-His | + | 11907 | 11976 | 70 | 0 |
| tRNA-Ser | + | 11977 | 12036 | 60 | 1 |
| tRNA-Leu | + | 12038 | 12107 | 70 | 0 |
| ND5 | + | 12108 | 13928 | 1821 | -17 |
| ND6 | - | 13912 | 14439 | 528 | 0 |
| tRNA-Glu | - | 14440 | 14508 | 69 | 4 |
| CYTB | + | 14513 | 15652 | 1140 | 4 |
| tRNA-Thr | + | 15657 | 15725 | 69 | -1 |
| tRNA-Pro | - | 15725 | 15790 | 66 | 0 |
| D-loop | + | 15791 | 16318 | 528 | 0 |

The intergenic nucleotides column indicates the number of intergenic or overlapping nucleotides ("-n" indicates overlapping regions).
