# Supplementary material for: Hunting the Extinct Steppe Bison (Bison priscus) Mitochondrial Genome in the Trois-Frères Paleolithic Painted Cave
Source: PLoS One. 2015 Jun 17;10(6):e0128267. doi: 10.1371/journal.pone.0128267 (PMC4471230; doi:10.1371/journal.pone.0128267)
Supplement: S5 Table — The compendium was established by comparing SGE2seq to the genome sequences available for 33 modern Bison bison specimens. The column names are as in S4 Table. (DOCX) [file pone.0128267.s007.docx]

**S5 Table.** List of the polymorphisms that are specific to the genome of *Bison priscus*.

| Position | Feature | Strand | Positioncodon | Base priscus | Base bison | Sequencepriscus | Sequence bison | Amino acid priscus | Amino acid bison | Type |
| --- | --- | --- | --- | --- | --- | --- | --- | --- | --- | --- |
| 144 | D-loop | + | NA | T | C | ACTTT | ACCTT | NA | NA | t |
| 204 | D-loop | + | NA | A | G | TTATA | CTGTA,TTGTA | NA | NA | t |
| 461 | s-rRNA | + | NA | T | C | CTTTT | CTCTT | NA | NA | t |
| 740 | s-rRNA | + | NA | T | C | ATTAA | ATCAA | NA | NA | t |
| 807 | s-rRNA | + | NA | G | A | GTGAC | GTAAC | NA | NA | t |
| 1158 | s-rRNA | + | NA | T | C | ATTAA | ATCAA | NA | NA | t |
| 1473 | l-rRNA | + | NA | C | T | TACCC | TATCC | NA | NA | t |
| 1495 | l-rRNA | + | NA | A | G | AAATA | AAGCA | NA | NA | t |
| 1496 | l-rRNA | + | NA | T | C | AATAA | AGCAA | NA | NA | t |
| 2054 | l-rRNA | + | NA | T | C | CCTAA | CCCAA | NA | NA | t |
| 2562 | l-rRNA | + | NA | G | A | TTGAC | TTAAC | NA | NA | t |
| 2581 | l-rRNA | + | NA | T | A | AATAA | AAAAA | NA | NA | v |
| 2978 | l-rRNA | + | NA | A | G | CAACA | CAGCA | NA | NA | t |
| 3102 | ND1 | + | 3 | A | G | ATATT | ATGTT | Met | Met | t |
| 3114 | ND1 | + | 3 | T | C | AATAT | AACAT | Asn | Asn | t |
| 3301 | ND1 | + | 1 | A | G | CAACC | CAGCC | Thr | Ala | t |
| 3555 | ND1 | + | 3 | G | A | TCGGT | TCAGT | Ser | Ser | t |
| 3798 | ND1 | + | 3 | G | A | ATGAT | ATAAT | Met | Met | t |
| 3826 | ND1 | + | 1 | C | T | TTCTA | TTTTA | Leu | Leu | t |
| 3862 | ND1 | + | 1 | T | C | TCTAC | TCCAC | Tyr | His | t |
| 4047 | ND1 | + | 3 | A | G | CCACA | CCGCA | Pro | Pro | t |
| 4453 | ND2 | + | 3 | A | G | CAATC | CAGTC | Gln | Gln | t |
| 4733 | ND2 | + | 1 | C | T | CCCTA | CCTTA | Leu | Leu | t |
| 4754 | ND2 | + | 1 | G | A | TAGTT | TAATT | Val | Ile | t |
| 4852 | ND2 | + | 3 | C | T | TACAA | TATAA | Tyr | Tyr | t |
| 5107 | ND2 | + | 3 | C | T | TTCAT | TTTAT | Phe | Phe | t |
| 5253 | ND2 | + | 2 | C | T | AGCCG | AGTCG | Ala | Val | t |
| 5291 | ND2 | + | 1 | C | T | TACTA | TATTA | Leu | Leu | t |
| 5374 | intergene | NA | NA | T | C | TGTTA | TGCTA | NA | NA | t |
| 5614 | tRNA-Cys | - | NA | A | G | AGAGC | AGGGC | NA | NA | t |
| 6283 | COX1 | + | 3 | C | T | CTCCC | CTTCC | Leu | Leu | t |
| 6301 | COX1 | + | 3 | C | T | GGCAT | GGTAT | Gly | Gly | t |
| 6469 | COX1 | + | 3 | T | C | TATTC | TACTC | Tyr | Tyr | t |
| 6634 | COX1 | + | 3 | C | A | ACCGG | ACAGG | Thr | Thr | v |
| 7382 | COX2 | + | 3 | T | C | TATCC | TACCC | Tyr | Tyr | t |
| 8245 | ATP8 | + | 3 | A | T | CTAAC | CTTAC | Leu | Leu | v |
| 8475 | ATP6 | + | 3 | T | C | AATCC | AACCC | Asn | Asn | t |
| 8748 | ATP6 | + | 3 | T | C | CCTAT | CCCAT | Pro | Pro | t |
| 8925 | ATP6 | + | 3 | T | C | TATGT | TACGT | Tyr | Tyr | t |
| 9545 | COX3 | + | 3 | C | T | GTCTA | GTTTA | Val | Val | t |
| 9632 | COX3 | + | 3 | T | C | CGTCA | CGCCA | Arg | Arg | t |
| 9713 | COX3 | + | 3 | C | T | GTCTG | GTTTG | Val | Val | t |
| 9867 | ND3 | + | 3 | T | C | ACTCT | ACCCT | Thr | Thr | t |
| 9909 | ND3 | + | 3 | G | A | GTGTA | GTATA | Val | Val | t |
| 10125 | ND3 | + | 3 | C | T | AGCCT | AGTCT | Ser | Ser | t |
| 10225 | tRNA-Arg | + | NA | C | T | AACTC | AATTC | NA | NA | t |
| 10496 | ND4L | + | 3 | C | T | GGCAC | GGTAC | Gly | Gly | t |
| 10692 | ND4 | + | 2 | C | T | AACAT | AATAT | Thr | Met | t |
| 10828 | ND4 | + | 3 | T | C | ATTTT | ATCTT,ATCTC | Ile | Ile | t |
| 10903 | ND4 | + | 3 | A | G | ACACT | ACGCT | Thr | Thr | t |
| 11153 | ND4 | + | 1 | T | C | CATTA | CACTA | Leu | Leu | t |
| 11305 | ND4 | + | 3 | C | T | TACCC | TATCC | Tyr | Tyr | t |
| 11324 | ND4 | + | 1 | C | T | CCCTA | CCTTA | Leu | Leu | t |
| 11551 | ND4 | + | 3 | C | T | ACCAT | ACTAT | Thr | Thr | t |
| 11581 | ND4 | + | 3 | T | C | CTTCT | CTCCT | Leu | Leu | t |
| 12192 | ND5 | + | 1 | C | T | AACCT | AATCC | Pro | Ser | t |
| 12194 | ND5 | + | 3 | T | C | CCTTC | TCCTC | Pro | Ser | t |
| 12272 | ND5 | + | 3 | C | T | ATCCA | ATTCA | Ile | Ile | t |
| 12338 | ND5 | + | 3 | C | T | TCCCT | TCTCT | Ser | Ser | t |
| 12488 | ND5 | + | 3 | C | T | ACCAT | ACTAT | Thr | Thr | t |
| 12542 | ND5 | + | 3 | A | G | GAAGG | GAGGG | Glu | Glu | t |
| 12701 | ND5 | + | 3 | A | T | CTACA | CTTCA | Leu | Leu | v |
| 12744 | ND5 | + | 1 | T | C | CCTTG | CCCTG | Leu | Leu | t |
| 12863 | ND5 | + | 3 | G | A | ATGGT | ATAGT | Met | Met | t |
| 13040 | ND5 | + | 3 | C | T | GGCCT | GGTCT | Gly | Gly | t |
| 13373 | ND5 | + | 3 | C | T | TACAG | TATAG | Tyr | Tyr | t |
| 13502 | ND5 | + | 3 | G | A | GGGTA | GGATA | Gly | Gly | t |
| 13650 | ND5 | + | 1 | C | T | ACCAC | ACTAC,ACTAT | His | Tyr | t |
| 13680 | ND5 | + | 1 | C | T | CCCTG | CCTTG | Leu | Leu | t |
| 13709 | ND5 | + | 3 | T | C | CATCG | CACCG | His | His | t |
| 13724 | ND5 | + | 3 | C | T | TACAT | TATAT | Tyr | Tyr | t |
| 13868 | ND5 | + | 3 | T | C | TATTT | TACTT | Tyr | Tyr | t |
| 13898 | ND5 | + | 3 | T | C | ATTAG | ATCAG | Ile | Ile | t |
| 14242 | ND6 | - | 3 | C | T | ACCAC | ACTAC | Val | Val | t |
| 14350 | ND6 | - | 3 | C | T | AACCC | AATCC | Gly | Gly | t |
| 14536 | CYTB | + | 3 | C | T | CACCC | CATCC | His | His | t |
| 14623 | CYTB | + | 3 | A | G | CTAGG | CTGGG | Leu | Leu | t |
| 14713 | CYTB | + | 3 | A | C | GCACA | GCCCA | Ala | Ala | v |
| 14989 | CYTB | + | 3 | T | C | AATTT | AACTT | Asn | Asn | t |
| 15200 | CYTB | + | 1 | T | C | TCTTA | TCCTA | Leu | Leu | t |
| 15894 | D-loop | + | NA | A | G | TTACA | TTGCA,CTGCA | NA | NA | t |
| 15951 | D-loop | + | NA | C | T | AACGT | AATGT,AATAT | NA | NA | t |
| 16034 | D-loop | + | NA | G | T | TAGTT | TATCC | NA | NA | v |
| 16035 | D-loop | + | NA | T | C | AGTTC | ATCCC,ATCCT | NA | NA | t |
| 16036 | D-loop | + | NA | T | C | GTTCC | TCCTC,TCCCC | NA | NA | t |
| 16063 | D-loop | + | NA | A | G | AAATT | AAGTT | NA | NA | t |
| 16072 | D-loop | + | NA | C | T | ATCGT | ATTGT | NA | NA | t |
| 16185 | D-loop | + | NA | T | C | ACTCC | ACCCC,ATCCC | NA | NA | t |
| 16209 | D-loop | + | NA | G | A | TGGAT | TGAAC | NA | NA | t |
| 16211 | D-loop | + | NA | T | C | GATTG | AACCG | NA | NA | t |
| 16212 | D-loop | + | NA | T | C | ATTGT | ACCGT | NA | NA | t |
| 16274 | D-loop | + | NA | T | C | CATCT | CACCT | NA | NA | t |
| 16284 | D-loop | + | NA | T | C | CGTCC | CGCCC,CGCTC | NA | NA | t |

The compendium was established by comparing SGE2seq to the genome sequences available for 33 modern *Bison bison* specimens. The column names are as in Table S4.
